# Image-Guided Robotic Radiosurgery for the Management of Intramedullary Spinal Cord Metastases—A Multicenter Experience

**DOI:** 10.3390/cancers13020297

**Published:** 2021-01-15

**Authors:** Felix Ehret, Carolin Senger, Markus Kufeld, Christoph Fürweger, Melina Kord, Alfred Haidenberger, Paul Windisch, Daniel Rueß, David Kaul, Maximilian Ruge, Christian Schichor, Jörg-Christian Tonn, Alexander Muacevic

**Affiliations:** 1 Charité–Universitätsmedizin Berlin, Corporate Member of Freie Universität Berlin, Humboldt-Universität zu Berlin, and Berlin Institute of Health, Department of Radiation Oncology, 13353 Berlin, Germany; carolin.senger@charite.de (C.S.); melina.kord@charite.de (M.K.); david.kaul@charite.de (D.K.); 2 European Cyberknife Center, 81377 Munich, Germany; markus.kufeld@cyber-knife.net (M.K.); Christoph.fuerweger@cyber-knife.net (C.F.); alfred.haidenberger@cyber-knife.net (A.H.); paul.windisch@ksw.ch (P.W.); alexander.muacevic@cyber-knife.net (A.M.); 3 Charité–Universitätsmedizin Berlin, Corporate Member of Freie Universität Berlin, Humboldt-Universität zu Berlin, and Berlin Institute of Health, Charité CyberKnife Center, 13353 Berlin, Germany; 4 Department of Stereotaxy and Functional Neurosurgery, University Hospital Cologne, 50937 Cologne, Germany; daniel.ruess@uk-koeln.de (D.R.); maximilian.ruge@uk-koeln.de (M.R.); 5 Department of Radiation Oncology, Kantonsspital Winterthur, 8400 Winterthur, Switzerland; 6 Department of Neurosurgery, Ludwig-Maximilians-University Munich, 81377 Munich, Germany; christian.schichor@med.uni-muenchen.de (C.S.); joerg.christian.tonn@med.uni-muenchen.de (J.-C.T.)

**Keywords:** radiosurgery, SBRT (stereotactic body radiation therapy), CyberKnife, robotic radiosurgery, intramedullary metastasis, neurooncology

## Abstract

**Simple Summary:**

Due to recent medical advancements, patients suffering from metastatic cancer have a prolonged life expectancy compared to several decades ago. Thus, the number of patients who experience metastasis to the spinal cord is increasing. Intramedullary metastases bear a dismal prognosis and cause considerable morbidity. Limited data are available on the treatment of such lesions. As surgery may be the mainstay of treatment for resectable and localized metastatic spread, previous case reports and series suggest radiosurgery to be a treatment alternative. This first multicenter study analyzes the efficacy of robotic radiosurgery (RRS) for the management of intramedullary metastases. Outcomes provide evidence that RRS is a safe, time-saving and effective treatment modality, especially for patients with unresectable lesions. Most patients die from systemic disease progression, while the majority of treated lesions remain controlled until death. Most symptoms improve or stay stable after treatment. These findings may guide further palliative care of affected patients.

**Abstract:**

Background: Intramedullary metastases are rare and bear a dismal prognosis. Limited data are available on the treatment of such lesions. As surgery may be the mainstay of treatment for patients with resectable and localized metastatic spread, previous case reports and case series suggest radiosurgery to be another viable treatment modality. This multicenter study analyzes the efficacy and safety of robotic radiosurgery (RRS) for intramedullary metastases. Methods: Patients who received RRS for the treatment of at least one intramedullary metastasis were included. Results: Thirty-three patients with 46 intramedullary metastases were treated with a median dose of 16 Gy prescribed to a median isodose of 70%. The local control was 79% after a median follow-up of 8.5 months. The median overall survival (OS) was 11.7 months, with a 12- and 24-month OS of 47 and 31%. The 12-month progression-free survival was 42% and at 24 months 25%. In addition, 57% of patients showed either an improved or stable neurological function after treatment delivery. Systemic disease progression was the main cause of death. No significant treatment-related toxicities were observed. Conclusions: RRS appears to be a safe, time-saving and effective treatment modality for intramedullary metastases, especially for patients with unresectable lesions and high burden of disease.

## 1. Introduction

Primary and secondary intramedullary tumors are rare, challenging to treat and often account for considerable morbidity. Their prognosis mainly depends on grading, location, size and resectability [1,2,3]. The most common primary intramedullary tumors include astrocytomas, ependymomas and hemangioblastomas. These three tumor entities account for more than 90% of all primary intramedullary tumors [1,2]. In contrast, secondary intramedullary lesions only account for a small proportion of spinal cord tumors [4]. Despite the rarity of intramedullary metastases, recent advances in imaging and therapy options as well as improved availability of magnetic resonance imaging (MRI) have led to an increasing incidence of secondary lesions [5]. Nevertheless, only less than 0.5% of cancer patients will be clinically affected by intramedullary metastases and only 0.6% of all spinal cord tumors are secondary due to metastatic disease [6,7,8]. Cases of metastatic intramedullary spread have been reported for a variety of cancer entities including lung, breast, renal cell, colorectal, ovarian and prostate cancer, as well as melanoma, but also rarer ones such as carcinoid tumors or thyroid cancer [6,9,10,11,12].

Secondary intramedullary lesions are linked with dismal median overall survival times of 3.5 to 7.3 months [6,13,14]. Given the scarcity of intramedullary metastases, high-level evidence and guidelines on their management are lacking. Current treatment options include microsurgical resection, chemotherapy, fractionated radiotherapy and stereotactic radiosurgery (SRS) as well as a combination of them [2,15,16,17]. In recent years, several studies have been published on the efficacy of surgery and radiotherapy for local control and their effect on neurological deficits [5,18,19,20,21]. Notably, sample sizes for the existing reports were limited. Furthermore, only limited data are available on the use of SRS as well as image-guided robotic radiosurgery (RRS) for intramedullary metastases. So far, a few studies, mostly case reports, have reported outcomes with this technique [19,21,22,23,24,25,26]. Moreover, no multicenter trials are available for this patient group. Herein, we describe the most extensive study on intramedullary spinal cord metastases treated with SRS to date in the framework of a retrospective multicenter study. The objective of this work is to describe the clinical outcomes of SRS utilizing RRS for the treatment of secondary intramedullary tumors as well as to compare our findings to the existing literature.

## 2. Results

### 2.1. Patient and Treatment Characteristics

A total of 33 patients with 46 secondary intramedullary tumors were included in this analysis. For seven patients, no dedicated clinical follow-up regarding the intramedullary lesion was obtainable. Four patients with four lesions without radiographic follow-up died within two months after treatment delivery. Twenty-nine patients with 42 lesions had in-house or external radiographic follow-up imaging available. The median and mean follow-up were 8.5 and 13.6 months, respectively. The median Karnofsky Performance Status (KPS) before treatment was 60%. The mean age at RRS was 48.4 years and the majority of patients were female (73%). Most metastases originated from breast cancer (48%), lung cancer (12%) and malignant melanoma (9%). The remaining entities included ovarian cancer, salivary duct carcinoma, colorectal cancer or sarcoma. 

In total, 41 of the 46 lesions (89%) received RRS as their primary treatment modality. One metastasis was partially resected, two were treated with intrathecal chemotherapy and two received a fractionated radiotherapy. All of these lesions received RRS as their secondary treatment. Most tumors were located in the thoracic spine (43%), followed by the cervical (33%) and lumbar spine (24%). Twenty-three patients (69%) had other central nervous system (CNS) metastases at the time of treatment delivery. The second, third and fourth most common metastatic sites were the lung (14 patients), bones (11 patients) and the liver (7 patients). Neurological deficits were present in 30 patients (91%) before RRS. Either motor or sensory dysfunctions of various degrees were present in 79% and both combined in 66% of patients. Fourteen patients (42%) were non-ambulatory at the time of RRS due to complete or incomplete paraplegia and tetraplegia. The median dose was 16 Gy, which was prescribed to a median isodose line of 70%. One patient received six Gy due to multiple previous radiotherapies in the affected area and the imminent palliative setting. The median tumor volume treated was 0.7 cc. Median conformity and heterogeneity indexes were 1.1 and 1.4, respectively. A total of three lesions in three patients were treated with three fractions to respect previous irradiations (3 × 5 Gy, 3 × 6 Gy and 3 × 7 Gy). All other patients underwent RRS with one fraction. The baseline characteristics are summarized in Table 1.

### 2.2. Outcome and Survival Data

**Figure 1:**
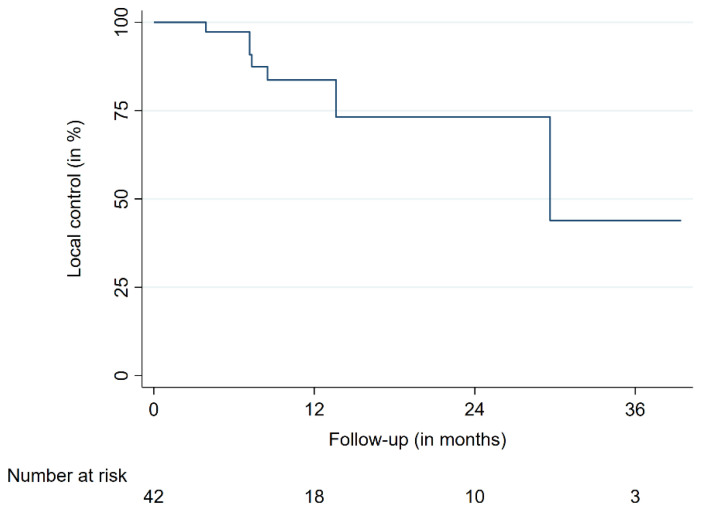
Local control.

At the last available follow-up, nine of the 42 lesions (21%) showed progression, whereas 33 remained controlled, leading to a local control (LC) rate of 79% after RRS. The nine progressive lesions were present in four patients, two suffering from breast cancer, one from malignant hemangiopericytoma and one from a peripheral primitive neuroectodermal tumor. The LC rates after 12 and 24 months were 84% and 73%, respectively (Figure 1). Regarding the clinical status, nine patients (27%) showed clinical improvement at their last follow-up, whereas ten (30%) did not show any significant changes. Two patients (6%) who were non-ambulatory before treatment partly recovered and became ambulatory with assistance. Seven (21%) patients showed a worsened neurological function, which was either related to a progression of their paraplegia or existing CNS metastases, including leptomeningeal disease. Two of these patients were suffering from a local treatment failure which caused subsequent clinical deterioration. All seven patients (21%) without specific clinical information concerning their intramedullary metastasis experienced overall worsening of their clinical status and cancer-associated symptoms. Dedicated and detailed information on pain relief due to the RRS treatment alone was not available as patients received intensive treatment with analgesics. 

**Figure 2:**
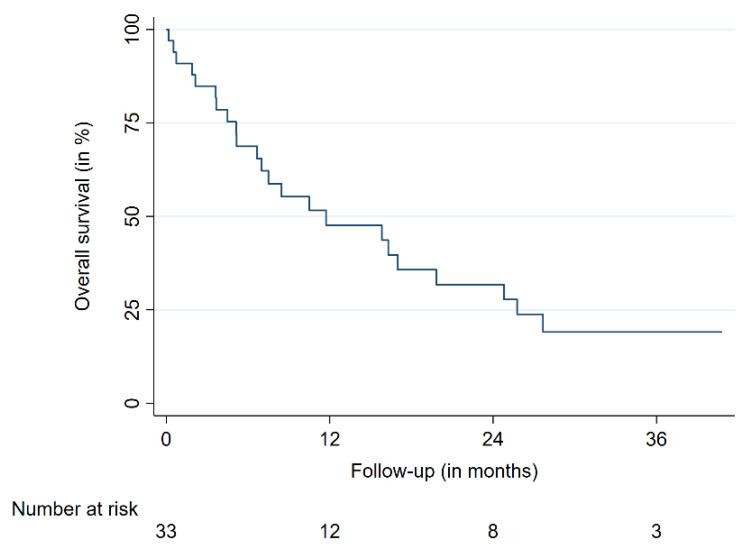
Overall survival.

**Figure 3:**
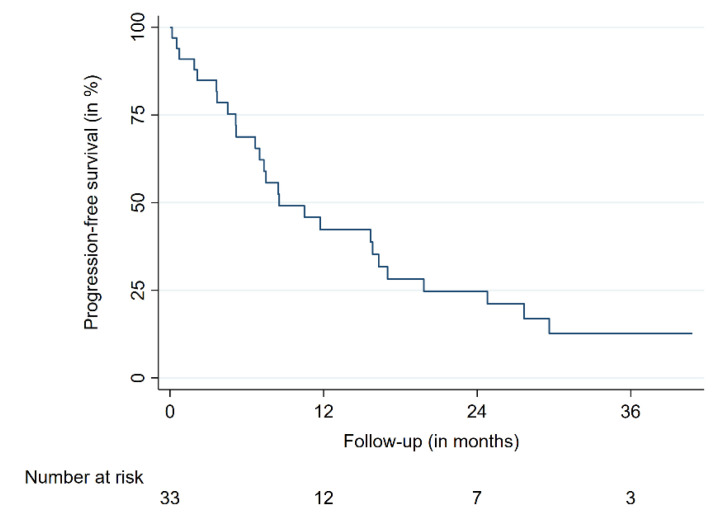
Progression-free survival.

The 12-month overall survival (OS) was 47%, the 24-month OS was 31%. The 12- and 24-month progression-free survival (PFS) were 42 and 25%, respectively (Table 2, Figure 2 and Figure 3). The median OS time was 11.7 months for all patients. Median OS times for breast cancer patients and the remaining patients were 17.0 and 6.6 months, respectively. No differences in patient characteristics between LC cases and local treatment failures have been identified (Table 2). Moreover, no significantly varying OS between patients suffering from breast cancer and other tumor entities was identified. A similar finding was present when patients with a single intramedullary lesion were compared to patients with multiple intramedullary lesions. One patient died from cancer-unrelated causes (respiratory failure secondary to fulminant pneumonia); the rest of the reported deaths were caused by systemic disease progression. No radiation necrosis, bleeding, myelopathy or other acute or delayed treatment-related complications or toxicities higher than grade 2 were observed.

## 3. Discussion

Despite recent advances in tumor therapy, intramedullary metastases represent a rare but considerable treatment challenge. Herein, we report the largest series of patients treated with SRS/RRS for secondary intramedullary tumors to date (Table 3). The objective of the study was to report a multicenter treatment experience to provide insights into the usefulness and efficiency of RRS in the setting of intramedullary metastases. Various studies on the role of surgery, radiotherapy and radiosurgery have been published so far, usually including case reports or analyzing a limited number of patients [5,13,14,19,21,22,27,28,29,30]. Moreover, immunotherapy may play a role in the treatment in the future [31]. While all these modalities on their own may play an essential role in the management of these lesions, a recent review showed favorable survival in patients receiving a multimodal treatment [17]. However, these results were only shown in patients with metastatic lung cancer, which limits the significance for intramedullary metastases of other tumors. 

Overall, three objectives can be considered particularly important for this patient group: local tumor control to prevent neurological deterioration, avoidance of unnecessary treatment-related morbidity as well as time-consuming treatment procedures in view of the imminent palliative setting. Several studies proposed and discussed early surgical resection for rapidly worsening patients who still show a decent performance status [4,6,14]. Usually, surgery is performed in patients not suffering from further metastases and where a histopathological diagnosis is needed [5,14]. If surgical resection is deemed achievable, it may be the primary treatment option for well-performing patients with a limited burden of disease and rapid neurological worsening. According to the sparse data available, LC after gross surgical resection is sufficient for the average life expectancy of affected patients but surgery may cause further neurological decline [5,32]. In contrast, the patient cohort not suitable for surgery due to performance status, widespread metastatic disease or unresectable lesions needs other treatment options. In this study with patients suffering from considerable further systemic disease, we report LC rates of 84% and 73% after 12 and 24 months. The considerable LC of intramedullary lesions achieved by RRS made systemic progression the main reason for the overall treatment failure and deterioration. Given the lack of reported LC rates due to the overall dismal prognosis and unavailability of follow-up imaging data, dedicated comparisons between the available treatment modalities are limited. This limitation underlines that available data are of retrospective nature, not standardized, and that patient samples of case reports and case series are particularly heterogeneous, especially considering their systemic burden of disease and performance status. As for the neurological deficits, 57% of patients in this study experienced either stable or improving symptoms, which is comparable to other SRS and RRS reports [21]. Moreover, surgery has also shown posttreatment improvements but may cause intermittent or persistent worsening and poses a greater complication risk compared to SRS and RRS [5,21,32]. 

Overall, the prevention of neurological deterioration is a major objective for this patient cohort, which was already expressed by other authors [14,30]. Moreover, and in contrast to the existing reports on RRS and SRS in general, the vast majority of patients (91%) in this multicenter study were treated in a single session [21]. As the imminent palliative setting must be taken into consideration when planning and choosing the treatment, reducing overall treatment time is an important point to consider not only when patients suffering from a high burden of disease are affected. Thus, treatments with RRS in one session may be a preferred option if applicable as it can help to stabilize the quality of life in the palliative setting given the favorable risk profile and time-saving treatment delivery.

While LC seems to be achievable in a considerable number of patients with most treatment modalities, secondary intramedullary tumors are linked with a dismal overall prognosis. OS is limited and many reports showed median survival times of less than six months [13,14,30,33]. Notably, the median OS for our cohort of patients with a high burden of disease was nearly three times as long as compared to the extensive cohort of Goyal et al. at Mayo Clinic (11.2 versus 3.6 months) [14]. Further studies also reported shorter median OS times [13,22,33]. One explanation for this finding is the recent improvement of systemic treatments, especially concerning immune- and targeted therapies, as our study cohort started in 2005 as compared to previous reports that also included patients treated before [13,14,22]. Moreover, our patients were primarily suffering from breast cancer and not lung cancer, which accounts for the majority of the OS difference, as patients with non-breast-cancer entities had a median survival of 6.6 months [6,14]. 

Overall, our patients were also younger in comparison to other case series and existing review data [6,13,14,22,34]. Systemic progression was the main cause of death, which is also contradictory to previous reports of Goyal et al. and Payer et al. [5,14]. However, this finding might be due to our patient selection, as the vast majority of patients were suffering from widespread metastatic disease, with 76% suffering from additional CNS lesions and lung metastases. Notably, other reports and most cases reported in the literature had less burden of systemic disease [14,30]. This may also explain this finding and respective differences. Besides these differences in patient characteristics and overall survival, treatment modalities must be carefully evaluated and chosen in regard to the patients’ condition, quality of life, tumor size and location as well as life expectancy. Nevertheless, the present study provides further evidence that SRS, especially RRS, could be used well in patients suffering from intramedullary metastases. This may be particularly important for patients with further metastatic spread and additional comorbidities preventing surgical tumor resection or other treatments. 

Finally, this study has inherent limitations due to its retrospective nature, sample size, lack of histopathological diagnosis in most cases and potential sampling biases, which all partially restrict further conclusions. Concerning the sample size and retrospective nature, one must acknowledge that patients with intramedullary metastases are still uncommon. Thus, prospective trials may be challenging to perform. Moreover, most patients today rather receive systemic palliative treatments than local therapies such as SRS or RRS. This is also reflected by our convenient sampling approach that may have led to respective sampling biases. Moreover, the lack of histopathological diagnosis is subordinate as all patients had at least one histopathological confirmation for their tumor diagnosis, with the respective primary tumor being the most evident explanation for development of intramedullary metastases. Finally, given the limited follow-up, some adverse radiation events such as radiation necrosis or myelopathy may have been missed or incorrectly classified as local failures.

## 4. Materials and Methods 

Thirty-three patients with 46 intramedullary metastases from three German treatment centers who were treated between June 2005 and June 2020 were included in this retrospective multicenter study. Diagnosis of intramedullary tumors was either confirmed by histology, radiographic appearance or both and validated by an interdisciplinary neurooncological tumor board involving neurosurgeons, neuroradiologists, neuropathologists and radiation oncologists. Medical history including pretreatments, neurological deficits, imaging data and histology was recorded prior to treatment. Decision for RRS treatment was made by the tumor board based on the available clinical data, present comorbidities and in accordance with the will and personal preferences of the respective patient. All patients underwent RRS using a CyberKnife^®^ robotic radiosurgery system (Accuray Inc., Sunnyvale, CA, USA). For treatment delivery, 1-mm thin-slice, contrast-enhanced computed tomography (CT) and 1-mm MRI scans (T1 gadolinium-enhanced) were acquired for every patient and subsequently merged for inverse treatment planning with a dedicated planning software (MultiPlan^®^, Precision^®^, Accuray Inc., Sunnyvale, CA, USA). For target definition, the visible tumor on contrast-enhanced imaging was contoured as the target volume, with no additional margin. The treatment dose was selected according to each center’s standard. Dose constraints were in accordance with TG101 if technically and medically appropriate and subject to modifications for individual cases [35]. Dose constraints were as follows: for single-fraction RRS, ≤0.35/≤ 1.2 cc of the spinal cord could receive 10.0/7.0 Gy, with a maximum point dose of 14.0 Gy in ≤0.35 cc; for three fractions of RSS, ≤0.35/≤ 1.2 cc of the spinal cord could receive 18.0/12.3 Gy, with a maximum point dose of 21.9 Gy in ≤0.35 cc. Adverse events (AE) were classified according to the Common Terminology Criteria for Adverse Events up to version 5.0. Radiation AE, local tumor response, clinical symptoms and adverse events were evaluated clinically and by imaging follow-up every three months for the first year, then every six months during follow-up or depending on the patients’ clinical status. Patients only undergoing biopsy for histological confirmation were classified as non-surgical cases. For the respective analyses on LC, every lesion was counted and analyzed separately. LC was defined as the absence of any tumor growth of the irradiated lesion on follow-up imaging (CT/MRI), whereas local treatment failure was defined as the absence of LC. OS, PFS and LC time were calculated from the time of RRS until death, progression or last follow-up according to the Kaplan–Meier method. Moreover, log-rank tests were used to compare survival times. Data were tested for normal distribution using the Shapiro–Wilk test and graphical appearance, including skewness and kurtosis. Normally distributed continuous variables were analyzed with the unpaired Student’s *t*-test, non-normally distributed data with Wilcoxon rank-sum tests. All *p*-values were two-sided and statistical significance was set at *p* ≤ 0.05. Statistical analyses were performed with STATA MP 16.0 (StataCorp, College Station, TX, USA). Various keywords were used to conduct a PubMed-based literature search to identify published reports on the use of SRS/RRS for intramedullary metastases. Only studies with full-body texts in English which reported the primary or secondary radiosurgical treatment with up to five fractions were reviewed.

## 5. Conclusions

RRS appears to be safe and efficient for the management of intramedullary metastases. Local tumor progression and symptom worsening were prevented in the majority of treated patients. Despite the limited sample size of this study, RRS can be considered as a primary non-invasive treatment option for unresectable or metastatic lesions in selected patients. In palliative care settings with worsening neurological deficits, RRS may prevent further clinical deterioration without the risks of time-consuming and invasive treatments.

## Figures and Tables

**Table 1:** Patient and treatment characteristics.

Patient and Treatment Characteristics			
Total number of patients	33		
Total number of lesions	46		
Sex male (%)/female (%)	9 (27)/24 (73)		
	Median	Mean	Range
Age (years)	48.4	49.2	11.3–74.4
Pretreatment KPS (%)	60	65.4	30–90
Follow-up (months)	8.5	13.6	1–40.8
Tumor volume (cc)	0.7	1.1	0.1–5.8
Dose (Gy)	16	16.1	6–24
Fractions	1	1.1	1–3
Prescription isodose (%)	70	69.0	60–80
Conformity index	1.1	1.2	1–1.6
Homogeneity index	1.4	1.4	1.2–1.67
Coverage	95	92.5	71.4–99.8
Tumor location	Cervical	Thoracic	Lumbar
Number of lesions (%)	15 (33)	20 (43)	11 (24)
Tumor entities			
Histology	Number of patients		
Breast (%)	16 (48)		
Lung (%)	4 (12)		
Malignant melanoma (%)	3 (9)		
Other (%)	10 (31)		

cc = cubic centimeter, Gy = Gray, KPS = Karnofsky Performance Status.

**Table 2:** Comparison between LC cases and local treatment failures with outcome and survival rates.

Comparison of Locally Controlled and Uncontrolled Patients				
Variable		Local control	Treatment failure	*p*-value
		Mean (±SD)		
Age		49.1 (11.2)	48.8 (11.5)	0.94
Tumor volume (cc)		1.1 (0.9)	0.8 (0.4)	0.30
Dose (Gy)		15.7 (2.2)	16.2 (0.9)	0.56
Prescription dose		69.0 (4.4)	68.8 (3.3)	0.67
Conformity index		1.2 (0.1)	1.2 (0.1)	0.63
Homogeneity index		1.4 (0.1)	1.4 (0.1)	0.69
Coverage (%)		91.9 (7.7)	94.7 (4.8)	0.31
Outcome and Survival				
Variable	Time (in months)	Value (%)	95% Confidence interval (%)	
LC	12	84.6	66.9–93.2	
	24	73.3	50.3–86.9	
PFS	12	42.8	25.5–59.0	
	24	25.0	11.3–41.3	
OS	12	47.5	29.3–63.7	
	24	31.6	15.7–48.9	

cc = cubic centimeter, Gy = Gray, LC = local control, PFS = progression-free survival, OS = overall survival.

**Table 3:** Literature review of intramedullary metastases primarily or secondarily treated with SRS.

Author	Year	Number of Patients (Metastases)	Number of Primarily Treated Metastasis	Treatment Modality	Median Follow-Up in Months	Dose/Fractions	Clinical Outcome	Radiographic Outcome
Garcia et al. [27]	2016	1 (1)	1	CK	37	14 Gy in 1 fraction	Stable	LC 100%
Veeravagu et al. [22]	2012	9 (11)	11	CK	NR (median survival: 4.1 months)	Median: 21 Gy in 3 fractions	Improvement: 9% Stable: 36% NA: 54%	LC 100% in four patients with follow-up imaging.
Lieberson et al. [9]	2012	1 (1)	0	CK	3	27 Gy in 3 fractions	Stable	LC 100%
Parikh et al. [23]	2009	1 (1)	0	CK	26	15 Gy in 3 fractions	Improvement	LC 100%
Shin et al. [19] *	2009	9 (11)	8	LINAC	NR (median survival: 8 months)	Median: 14 Gy in 1 fraction	Improvement: 88%, Stable: 11%, Worse: 11%	LC 89% in eight patients with follow-up imaging.
Chamberlain et al. [25]	2010	1 (1)	1	CK	NR	NR	NR	NR
Barrie et al. [28]	2019	1 (1)	1	CK	26	25 Gy in 5 fractions	Worse	Progressive disease
This series	2020	33 (46)	41	CK	8.5	Median: 16 Gy in 1 fraction	Improvement: 27%, Stable: 30%, Worse: 21% of patients with available clinical follow-up.	LC 79% in 29 patients with follow-up imaging.

* = Series includes 4 intradural extramedullary lesions. CK = CyberKnife, Gy = Gray, NA = not available, NR = not reported, LINAC = linear accelerator.

## Data Availability

The data that support the findings of this study are available from the corresponding author, F.E., upon reasonable request.
